# Effect of exercise on postprandial lipaemia in children with sickle cell disease

**DOI:** 10.3389/fspor.2025.1560669

**Published:** 2025-05-07

**Authors:** Mahfoodha Al Kitani

**Affiliations:** Physical Education & Sport Sciences Department, College of Education, Sultan Qaboos University, Muscat, Oman

**Keywords:** sickle cell disease, postprandial lipemia, high-fat mixed meals sickle cell disease, triacylglycerols, exercise, endothelial dysfunction, cardiovascular risk, inflammation

## Abstract

Elevated plasma triacylglycerol (TAG) levels are associated with endothelial dysfunction, inflammation, and increased vascular complications, particularly in populations such as individuals with sickle cell disease (SCD). This study aimed to investigate the effect of a single exercise session on postprandial TAG levels in children with SCD following the consumption of a high-fat meal. The high-fat meal was used to induce a significant postprandial increase in plasma triayglecrol (TAG) levels, aknown risk factor for endothelial dysfunction and vascular implication, particualry in population with Sickle Cell Disease (SCD). Twelve male children with SCD (mean age: 10.5 ± 1.2 years) participated in two 2-day trials, one involving brisk walking (exercise trial) and one with rest (rest trial), in a randomized, counter-balanced design. A mixed high-fat test meal (97 g fat, 124 g carbohydrate, 1,450 kcal) was administered after a 10-h overnight fast, and blood samples were collected at baseline, 60, 120, 240, 300, and 360 min post-meal to measure serum TAG, glucose, insulin, and total cholesterol concentrations. Postprandial TAG levels significantly increased in both trials, but the exercise trial showed a significantly lower TAG response compared to the rest trial (*P* < 0.05). The area under the curve (AUC) for TAG was greater in the rest trial than in the exercise trial (*P* < 0.05). Specifically, TAG concentrations were significantly lower in the exercise trial at 120 and 180 min post-meal (*P* < 0.05), indicating faster clearance of TAG following exercise. Insulin and glucose concentrations also increased post-meal, with significantly lower insulin and glucose AUC values in the exercise trial compared to the rest trial (*P* < 0.05). No significant differences were observed in total cholesterol between the two trials at 360 min post-meal. These findings suggest that a single bout of exercise prior to a high-fat meal reduces postprandial TAG concentrations in children with SCD, consistent with previous studies in healthy populations. The reduction in postprandial TAG may have implications for improving endothelial function and reducing the risk of cardiovascular and vaso-occlusive events in individuals with SCD. Further studies are needed to explore the long-term effects of regular exercise on lipid metabolism and disease complications in this population.

## 1 Introduction

Elevated plasma triacylglycerol (TAG) levels have been reported to result in endothelial dysfunction in healthy individuals (1–4). Endothelial function is impaired in patients with sickle cell disease (SCD) (5–7), making a better understanding of TAG concentrations in this population important. Adults with SCD exhibit higher TAG, lower cholesterol, high-density lipoprotein (HDL), and low-density lipoprotein (LDL) concentrations than healthy subjects (8). Furthermore, elevated serum TAG levels in patients with SCD are associated with elevated markers of hemolysis, inflammation, and endothelial activation, indicating an association between TAG levels, vascular dysfunction, increased rate of hemolysis and a painful crisis (8–10). Despite lower serum cholesterol concentrations in SCD, LDL in patients with SCD is more prone to oxidation, resulting in the release of free radicals and oxidative stress, which contributes to subsequent injury to the endothelium (11, 12).

Postprandial lipemia is the presence of high concentrations of TAG in the blood after a meal (13). Postprandial lipemia, rather than the measurement of fasting TAG concentrations, is more informative when trying to understand changes in plasma TAG concentrations and is considered a more effective approach to studying lipid metabolism (14). The Columbia University Biomarkers Study showed that the postprandial TAG response in adolescents is similar to that in adults (15). Physical activity performed immediately before intake of high-fat meals have been shown to lower postprandial TAG in non-endurance-trained people (16, 17). Furthermore, several studies have examined the influence of physical activity performed the day before consuming a high-fat meal on postprandial TAG concentrations, with consistent findings indicating that consistent physical activity effectively lowers postprandial TAG concentrations (16, 18, 19). Tolfrey et al. (20) examined the effect of 30 and 60 min of jogging on postprandial TAG in 13-year-old boys, while Barrett et al. (19) investigated the effect of continuous-exercise and intermittent-games activity in 15-year-old boys. The results of both studies indicated that, regardless of the type and duration of exercise, postprandial TAG levels were significantly lowered. However, to date, no studies have examined the effect of exercise on postprandial TAG in individuals with SCD.

Lipid profiles can serve as an indicator for assessing the risk of cardiovascular and inflammatory complications in patients with Sickle Cell Disease (SCD). However, their ability to predict vaso-occlusive crises is limited, as lipid levels can be influenced by factors such as hemolysis and anemia, which are common in SCD. Therefore, lipid profile measurements should be used in conjunction with other clinical parameters to provide a more comprehensive evaluation of patient risk. Therefore, this study aimed to investigate the effect of high-fat mixed meals on postprandial TAG levels in children with SCD and to examine the effect of a single exercise session on postprandial TAG.

Given the effect of a high-fat meal on plasma lipids and the effect of exercise in postprandial TAG in non-SCD adolescents, we hypothesized that postprandial TAG will increase after a high-fat meal in children with SCD and that prior exercise will reduce the magnitude of the postprandial TAG response.

## 2 Materials and methods

### 2.1 Subjects

A total of 18 male children diagnosed with Sickle Cell Disease (SCD) were initially recruited from the Hematology and Children's Health Unit at Sultan Qaboos University Hospital (SQUH). However, due to various factors such as difficulties in transportation and tight parents schudels, only 12 participants successfully completed the study and were included in the final analysis. Subjects were admitted to the Pediatric unit at SQUH for testing. During admission, body composition and anthropometric measurements were conducted. Electrocardiograph was performed during exercise to ensure participant safety. Physical examination and medical history were carried out in the hospital for each patient before engaging in the study. Patinets with a history of stroke, serious cardiac arrhythmias, vaso-occlusion, hypertension, blood transfusion, or those taking medication were excluded from the study. A physician and staff nurse were present during all testing. The study protocol was approved by the Medical and Ethical Research Committee at SQUH. Before participation in the study, the participants and their parents were informed of the study procedures, potential risks, and benefits; and written informed consent was obtained.

### 2.2 Design

#### 2.2.1 Research design

This study employed a randomized crossover design, where each participant completed two 2day trials: a brisk walking trial, and a rest trial. The 2-day trial was used because skeletal muscle lipoprotein lipase (LPL) activity is thought to peak 8 h after exercise (21), and this enzyme facilitates the removal of TAG from the blood (22). Trials were carried out in a randomized counter-balanced order with a 7-day gap between trials.

#### 2.2.2 Preliminary test

Before the main trials, all participants performed a sub-maximal incremental treadmill test. A modified Bruce protocol was used to determine the walking speed for each participant. Participants were encouraged to give their best effort during the brisk walking on the treadmill. Heart rates and ECG were monitored continuously during the test to detect abnormalities. The test was designed to elicit 75% of the maximum recommended heart rate for this population.

#### 2.2.3 Main trials

##### 2.2.3.1 First Day

On the first day of each trial, the participants reported to the laboratory at 9:00 a.m. On arrival, the participants were asked to sit quietly for 10 min, and then a baseline resting blood sample was obtained. During the walking trial, the participants performed three 10-minute bouts of treadmill brisk walking. A 10-minute rest interval followed each bout such that the total exercise session lasted for 50 min. Walking speed was determined by the cardiologist using ECG readings, targeting 75% of the maximum recommended heart rate of 175 bpm for individuals with SCD (62). For the rest trial, the participants were asked to sit quietly and watch TV at the children's hall of the hospital. On leaving the laboratory, the participants were instructed to have an early evening meal and to rest for the remainder of the evening. Participants were asked to report their diet on the evening of day one of the first trial and to repeat the same diet on the evening of day one of the second trial.

##### 2.2.3.2 Second day

On the second day of each trial, the participants reported to the laboratory at 9:00 a.m. after a 10-h overnight fast (no food or drink except water). The participants rested for 10 min after their arrival at the laboratory. A cannula was then inserted into an antecubital vein, and a baseline blood sample was collected. The participants then consumed (under supervision) a mixed standardized test meal that was high in fat. The test meal consisted of croissants, butter, high-fat ice-cream, chocolate and potato crisps with a macronutrient composition per 2 m^2^ body surface area of 97 g fat, 124 g carbohydrate and 1,450 kcal (23). Body surface area was calculated using Haycock equation which was validated for infants, children and adults (24). Further blood samples were obtained at 60, 120, 240, 300 and 360 min after ingesting the meal. Participants rested quietly during this period.

### 2.3 Blood sampling

Blood samples were collected before the test meal was ingested and five times hourly in the postprandial period. Participants rested quietly during this period. Blood was collected using standard blood collecting sets and drawn into sterile tubes (EDTA). Serum was allowed to clot at room temperature for 20 min. Samples were then centrifuged at 1,500 g for 15 min, and the serum was removed before being frozen at −70 °C. Glucose levels were measured using enzymatic methods (Cobas Integra 800; Roche, Switzerland). The levels of other biochemical markers, serum TAG, total cholesterol, HDL, and LDL, were also measured by enzymatic methods (Cobas Integra 800; Roche, Switzerland). Serum insulin was determined using standard fluoroimmunoassays (AutoDELFIA, Finland). Serum C-reactive protein (CRP) was measured using competitive immunoassay (Roche Diagnostics, Germany).

### 2.4 Statistical analysis

Data were analyzed using SPSS version 17 (SPSS Inc., Chicago, USA). A two-way ANOVA with repeated measures was used to investigate differences between trials and across time for the following dependent variables: TAG, glucose, insulin, CRP, total cholesterol, LDL, and HDL. The area under curve (AUC) for TAG, glucose, and insulin was calculated using the trapezoid method. Significant interactions were identified by ANOVA and AUC, Bonferroni adjusted *t*-tests used for follow-up comparisons. Statistical significance was set at *P* ≤ 0.05. Data are presented as mean (SD).

## 3 Results

### 3.1 Triacylglycerols

**Figure 1:**
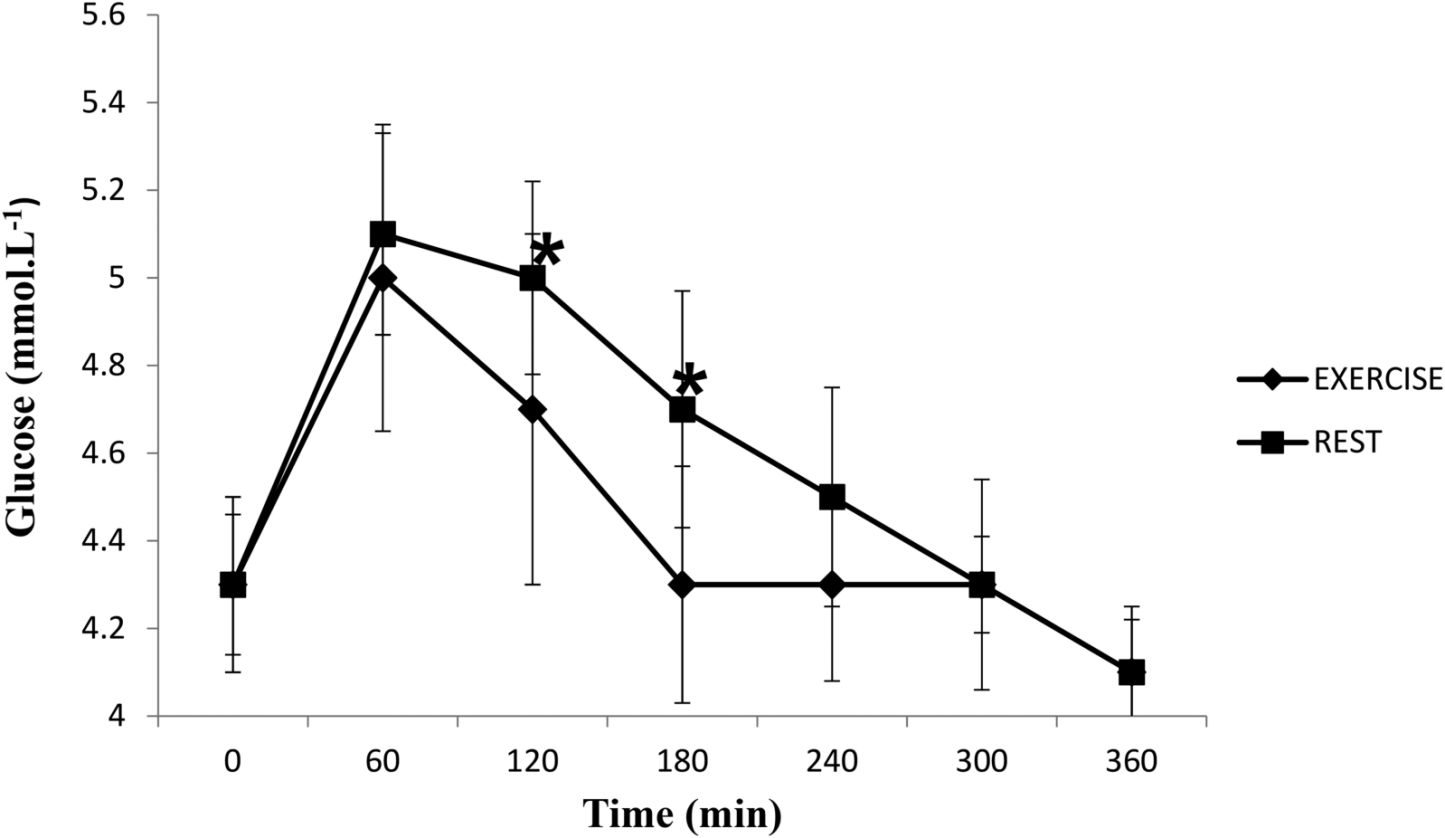
Mean (SD) TAG in the postprandial period following rest or exercise on the day before. *Significant difference between rest and exercise trials (*P* < 0.05).

**Figure 2:**
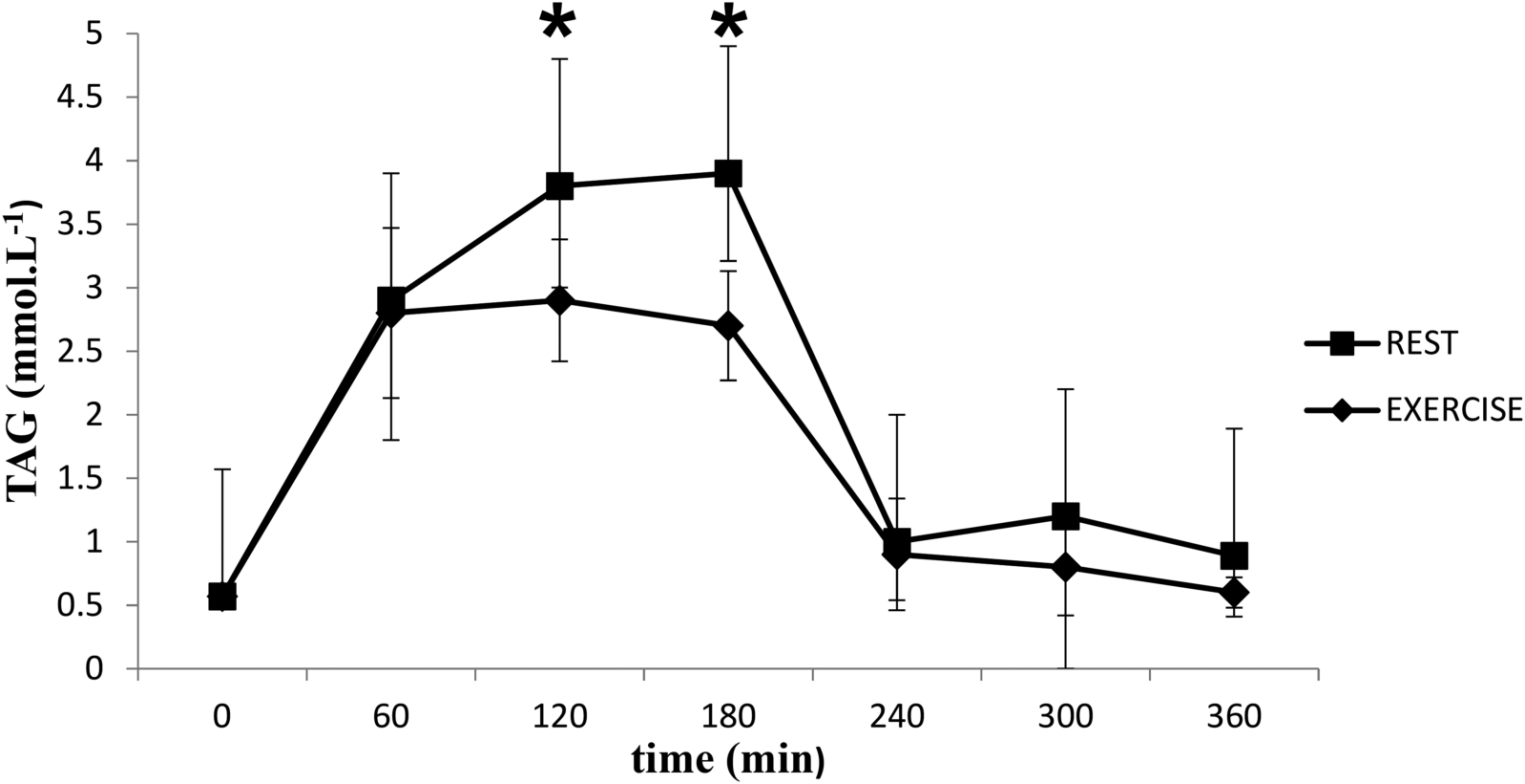
Mean (SD) TAG AUC in the postprandial period during rest and exercise trials. *Different from exercise trial (*P* < 0.05).

TAG concentrations increased following the meal (time effect, *P* < 0.05) and were higher in the rest trial than the exercise trial (*P* < 0.05) (Figure 1). TAG AUC for the rest trial was greater than TAG AUC for the exercise trial (*P* < 0.05) (Figure 2). There was a significant interaction effect, and *post hoc* analysis showed that TAG concentrations were significantly higher in the rest trial than the exercise trial 120 and 180 min after consuming the test meal.

### 3.2 Insulin

**Figure 3:**
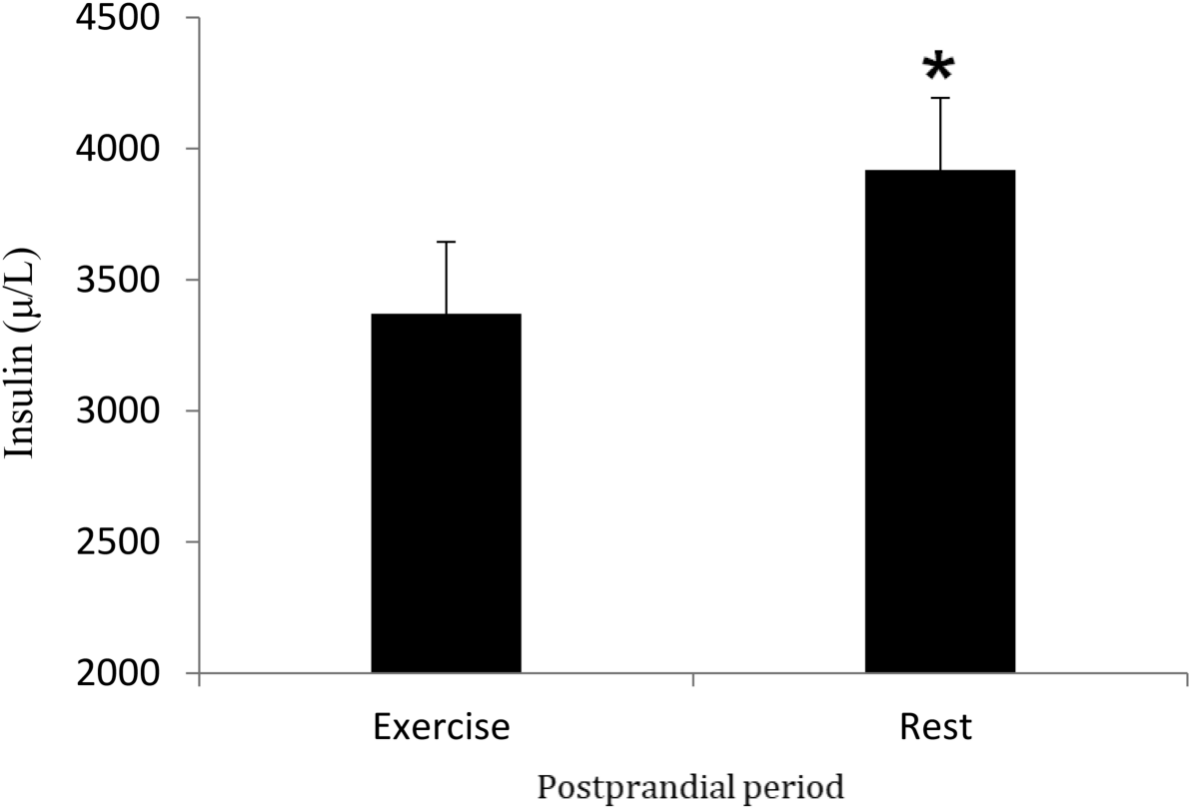
Mean (SD) insulin in the postprandial period following rest or exercise on the day before. *Significant difference between rest and exercise trials (*P* < 0.05).

**Figure 4:**
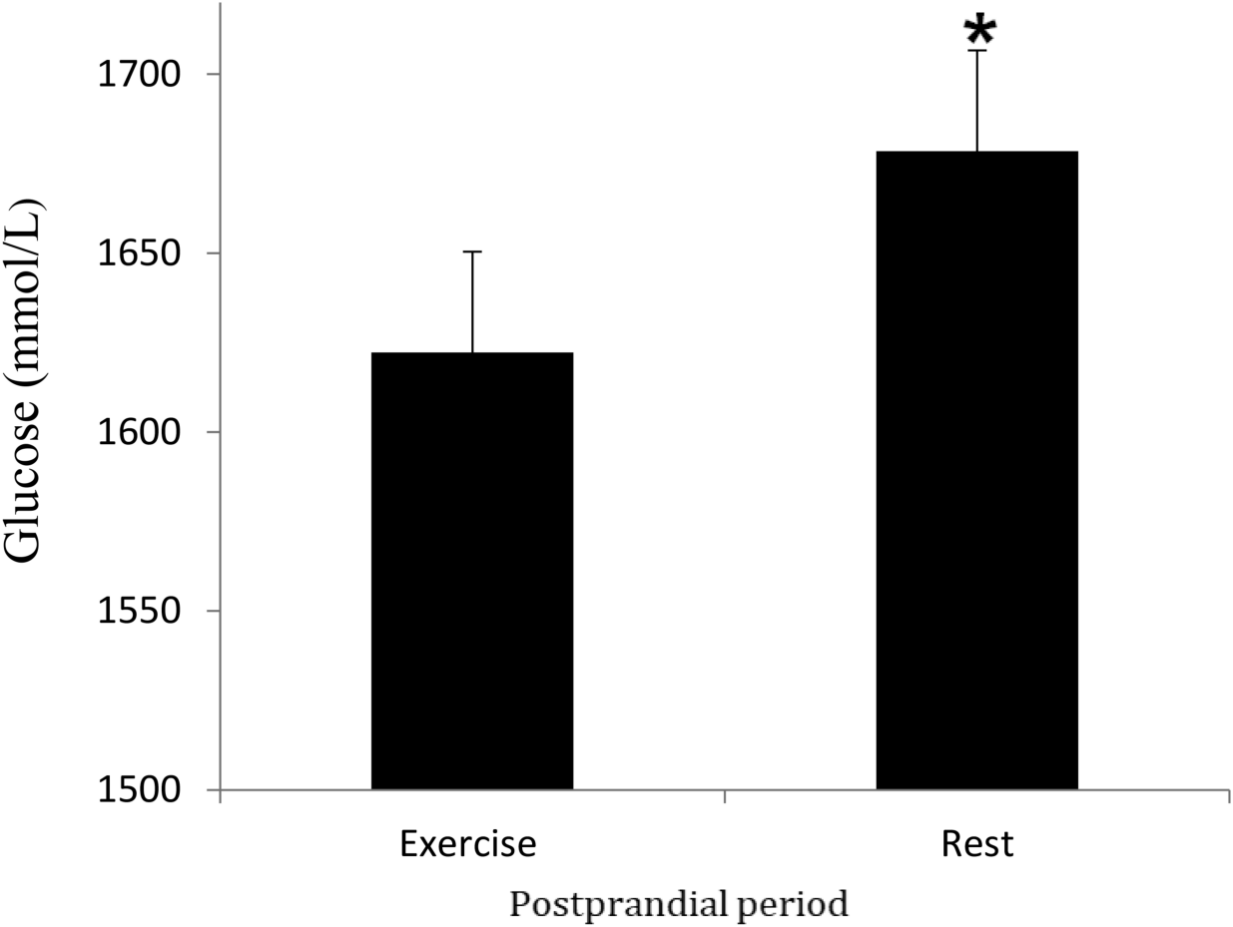
Mean (SD) insulin AUC in the postprandial period during rest and exercise trials. *Different from exercise trial (*P* < 0.05).

Insulin concentrations increased following the meal (time effect, *P* < 0.05) and were significantly higher in the rest trial than the exercise trial (*P* < 0.05) (Figure 3). Insulin AUC for the rest trial was greater than Insulin AUC for the exercise trial (*P* < 0.05) (Figure 4). There was a significant interaction effect, and *post hoc* analysis showed that insulin concentrations were significantly higher in the rest trial than the exercise trial 60, 120 and 180 min after consuming the test meal, and lower at 360 min.

### 3.3 Glucose

**Figure 5:**
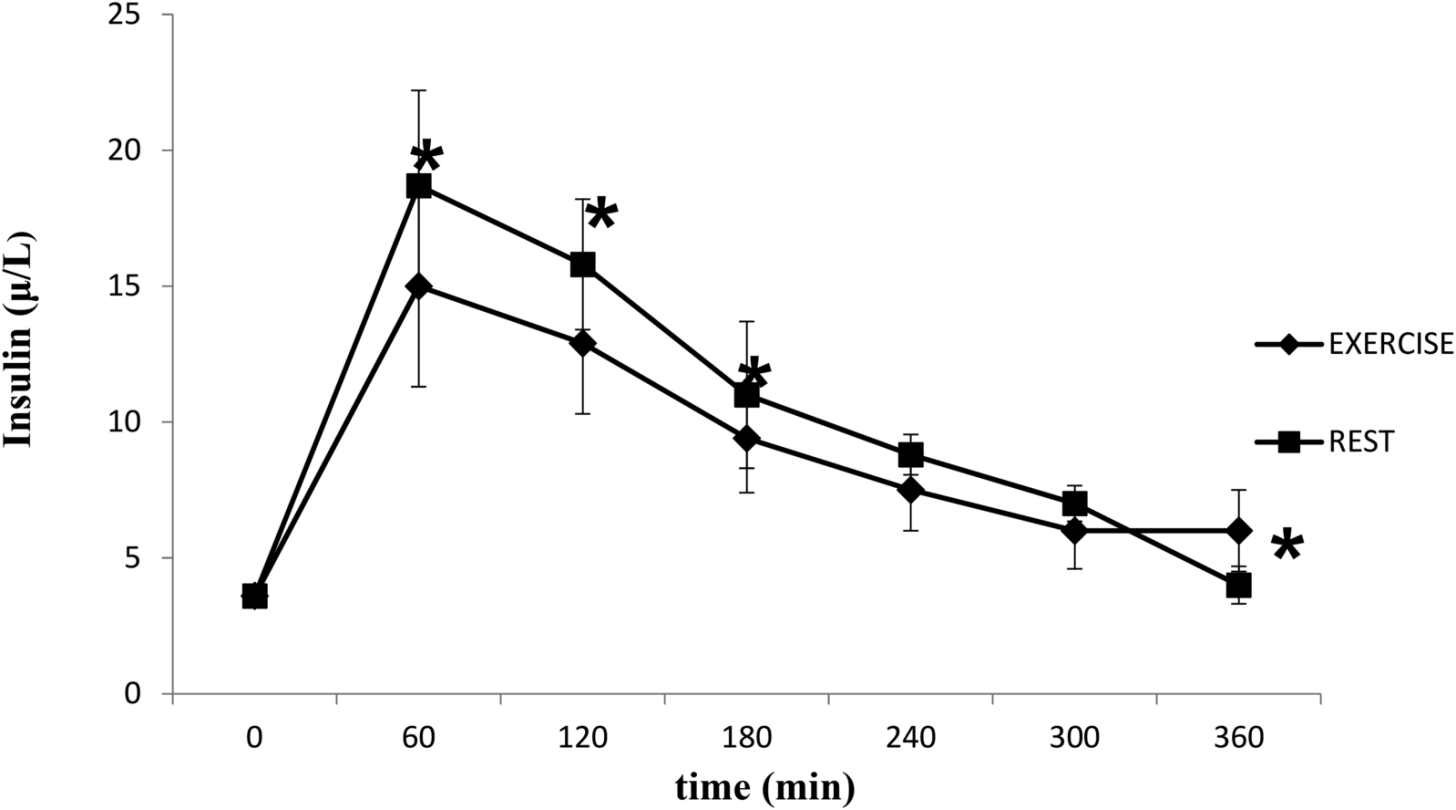
Mean (SD) glucose concentrations in the postprandial period following rest or exercise on the day before. *Significant difference between rest and exercise trials (*P* < 0.05).

**Figure 6:**
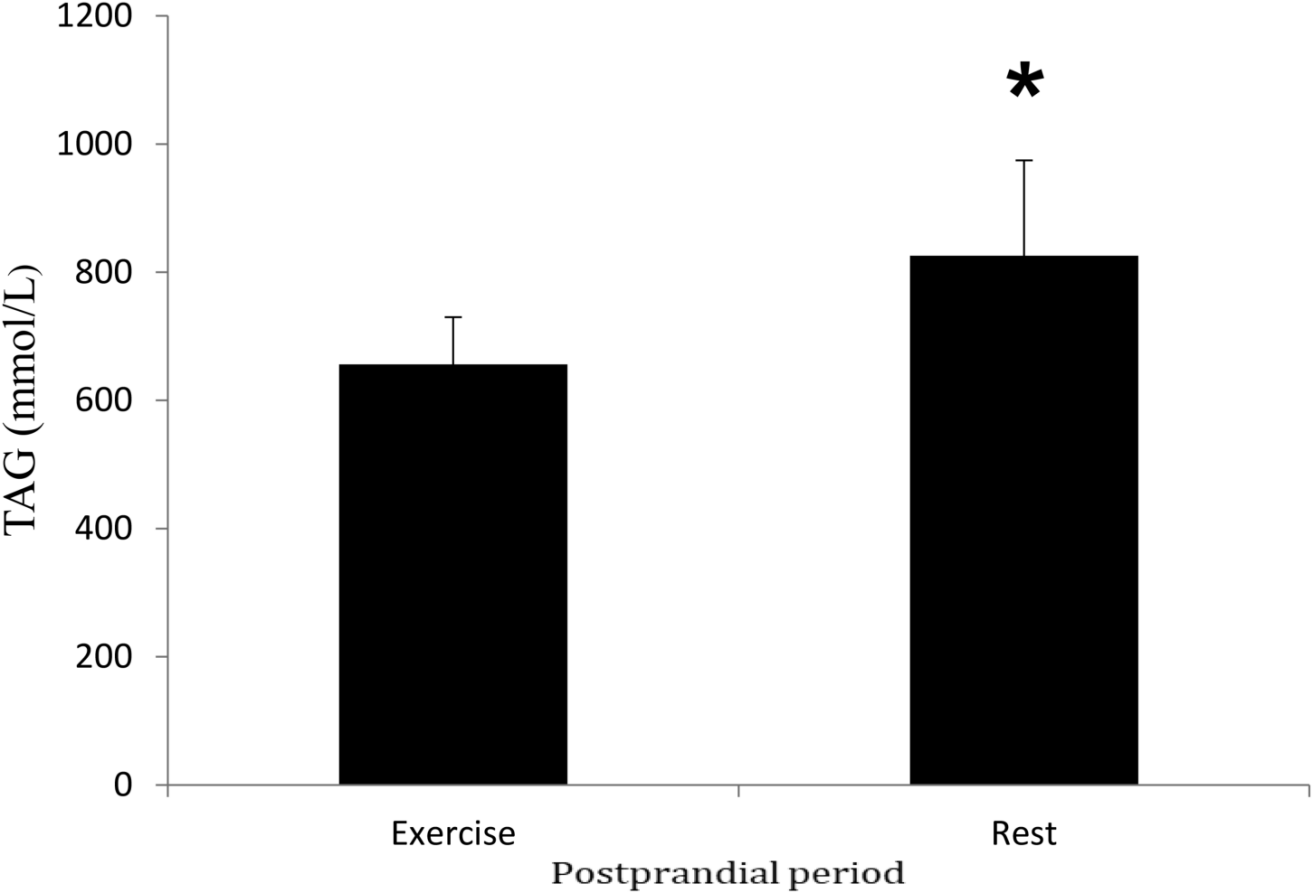
Mean (SD) glucose AUC in the postprandial period during rest and exercise trials. *Different from exercise trial (*P* < 0.05).

Glucose concentrations increased after the meal (time effect, *P* < 0.05) and were significantly higher in the rest trial than the exercise trial (*P* < 0.05) (Figure 5). Glucose AUC for the rest trial was greater than glucose AUC for the exercise trial (*P* < 0.05) (Figure 6). There was a significant interaction effect, and *post hoc* analysis showed that glucose concentrations were significantly higher in the rest trial than the exercise trial 120 and 180 min after consuming the meal.

## 4 Discussion

This study was designed to investigate the effect of consuming a high-fat mixed meal on postprandial TAG concentrations in Omani children with SCD and the effect of a single bout of exercise on this TAG response. The main findings were that a high-fat meal significantly increased plasma TAG concentrations and that postprandial TAG, insulin, and glucose responses were lower when exercise was performed the day before the meal, consistent with findings in healthy children (20, 25, 26). There was no difference in total cholesterol response between the exercise trial and the rest trial six hours after the meal.

Postprandial TAG is an important indicator for cardiovascular disease. The rise in plasma concentrations of TAG-rich lipoproteins following the consumption of a high-fat meal is considered a more reliable marker of future cardiovascular disease than fasting TAG levels alone (27, 28). Recently, the American Heart Association pointed to the need to design strategies aimed at primary prevention of atherosclerotic cardiovascular disease in childhood (29). In SCD, high serum TAG has been associated with impaired endothelial function, increase hemolysis, anemia, inflammation, and painful crisis (7, 30–32). To our knowledge, this is the first study that has assessed the effect of exercise on postprandial TAG in children with SCD. Therefore the finding that a single bout of exercise reduces postprandial plasma TAG concentrations in children with SCD is novel.

Physical activity is an effective tool for lowering postprandial TAG concentrations (33, 34). A single bout of low- and moderate-exercise intensity was found to lower postprandial TAG levels in healthy adults (17, 35, 36). Kashiwabara et al. (37) and Miyashita et al. (38) reported that 30 min of accumulated brisk walking in one session reduces postprandial plasma TAG concentrations and increases fat oxidation. In addition, several studies that examined the influence of exercise on postprandial TAG levels in adolescent boys reported a decrease in postprandial TAG in response to exercise (19, 20, 26, 39, 40). This study reported similar results, indicating that postprandial TAG concentrations were higher in the rest trial than in the exercise trial. Specifically, TAG concentrations at 120 and 180 min post-meal were higher in the rest trial, suggesting faster TAG clearance in the exercise trial. The mechanisms responsible for this include increased LPL activity, enhanced lipid mobilization, increased blood flow to muscles, insulin sensitivity, hormonal changes, and dietary factors. These mechanisms ensure that the body efficiently utilizes fat for energy and recovery (41). One possible explanation for the faster TAG clearance in the exercise trial is an increase in the activity of LPL, a key enzyme in the TAG metabolism pathway. Previous studies have shown that adults who exhibit an increase muscle activity or plasma LPL after a single bout of moderate exercise also exhibit a simultaneous reduction in fasting and postprandial TAG concentrations (18, 42). Recent studies have highlighted that lipoprotein lipase (LPL) plays a central role in TAG metabolism by hydrolyzing triglycerides in lipoproteins into free fatty acids and monoacylglycerol (43). However, it is also recognized that other mechanisms contribute to the reduction of TAG levels. For instance, increased activity of adipose LPL enhances the uptake and storage of fatty acids in adipose tissue, which further influences overall lipid metabolism (44). Additionally, reduced hepatic secretion of very low-density lipoproteins (VLDL) decreases the delivery of triglyceride-rich particles into circulation, thereby lowering plasma TAG levels (45).

Concentrations of insulin and glucose were higher at specific time points in the rest trial compared to the control trial, with a significantly greater insulin AUC in the rest trial. These findings demonstrate that carbohydrates in the mixed meal triggered an insulin response, which is consistent with previous literature on postprandial insulin dynamics. However, these results differ from those of Cockcroft et al. (46) and Buchan et al. (47), who reported no difference in insulin response to exercise in healthy adolescent boys, suggesting that factors such as age and physical activity status may influence the insulin response. Insulin plays a key role in regulating plasma TAG concentration by down-regulating LPL activity in skeletal muscle (43). Additionally, it has been shown that in adults, insulin decreases skeletal muscle LPL (48, 49). Therefore, the greater rise in insulin observed in the rest trial may, to some extent, explain the slower rate of TAG clearance, potentially linking altered insulin dynamics to lipid metabolism under different experimental conditions.

Fasting total cholesterol concentrations have been reported to be lower in patients with SCD than in healthy controls, suggesting a unique lipid metabolism in SCD that may have implications for cardiovascular health (10, 50, 51). Similar results were found in Nigerian boys and girls (63). The results of this study align with those of Dantas et al. (52), showing lower baseline total cholesterol in this population compared to healthy adolescent boys (Table 1). While MacEneaney et al. (23) reported higher total cholesterol six hours post-meal in the rest trial compared to the exercise trial, no significant difference in total cholesterol response was observed between the two trials in this study. Despite this, maintaining a favorable lipid profile, particularly HDL cholesterol, remains important for cardiovascular health, and exercise may still contribute to long-term improvements in HDL levels.

**Table 1:** Effect of acute exercise on cholesterol.

Variable	Rest		Exercise	
	0 h	6 h	0 h	6 h
Total Cholesterol (mmol L^−1^)	3.0 (.7)	3.3 (.9)*	2.8 (.6)	3.1 (.7)
LDL-Cholesterol (mmol L^−1^)	1.5 (.8)	—	1.7 (.5)	—
HDL-Cholesterol (mmol L^−1^)	1.2 (.5)	1.3 (.2)	1.0 (.3)	1.1 (.1)*
CRP (mg L^−1^)	6.5 (.9)	6.3 (.8)	5.7 (.5)	5.5 (.3)

Values are Mean (SD).

* Significant difference between rest and exercise trials (*P* < 0.05).

Postprandial TAG levels reduced through exercise utilized in this study setting, suggesting health advantages for this population. This reduction in TAG levels may contribute to improved metabolic control, which is particularly significant for individuals with SCD, who often face increased cardiovascular risk and inflammation. The lowering of postprandial TAG concentrations in individuals with SCD through exercise may have beneficial effects in reducing pain crisis episodes and disease complications due to reduced hemolysis, endothelial activation, and inflammation described earlier. These benefits highlight the potential of exercise as a therapeutic strategy to mitigate key aspects of SCD pathophysiology (8, 9, 53, 54). An elevated circulating CRP level is indicated to be a marker of vascular endothelial dysfunction (55, 56), as the accumulating evidence suggests that CRP induces expression of IL-6 and mediates LDL cholesterol uptake by endothelial macrophages (56–58). An increase in fasting and postprandial TAG has been indicated to be associated with higher fasting and postprandial CRP levels (59,60,61). In this study, there was no significant change in CRP concentrations six hours after consuming the meal in both trials. Elevated TAG concentrations are associated with increased hemolytic markers, activation of the endothelium, and inflammatory responses, leading to higher chances of vaso-occlusive and inflammatory events (8). TAG elevation is also associated with increased acetylcholine release leading to reduced blood flow to tissue (8). Therefore, any reduction in TAG concentrations is likely to have a beneficial impact on children with SCD. The net effect of reduction in TAG, insulin, and total cholesterol concentration resulting from mild- to moderate-exercise bouts may considerably reduce disease complications and enhance the quality of life for individuals with SCD, particularly if exercise is performed regularly.

## 5 Conclusion

This study demonstrates that a single bout of exercise before the consumption of a high-fat meal reduces postprandial TAG concentrations in children with sickle cell disease (SCD), echoing the results found in healthy children. While much of the existing literature on the effect of exercise on postprandial TAG levels has focused on adults, this study is the first to examine this response in children with SCD. The findings not only support previous reports on healthy children but also provide valuable insights into the potential role of exercise in reducing postprandial TAG levels in children with SCD.

However, the benefits of exercise may extend beyond metabolic improvements. Regular physical activity could strengthen hematological health by improving vascular function, reducing hyperviscosity, and promoting better blood flow, which are crucial factors in managing SCD. While this study primarily addresses the acute effects of exercise on TAG levels, it also highlights the need for further research to explore the long-term impact of exercise on both metabolic and hematological outcomes in individuals with SCDIn conclusion, while this study contributes to understanding the role of exercise in managing postprandial lipid levels in children with SCD, additional investigations are required to fully elucidate the long-term benefits of regular exercise on overall disease management, including hematological health.

## Data Availability

The original contributions presented in the study are included in the article/Supplementary Material, further inquiries can be directed to the corresponding author.
